# The Use of Autologous Blood Patch in Ullrich Muscular Dystrophy and Recurrent Pneumothorax

**DOI:** 10.7759/cureus.25961

**Published:** 2022-06-15

**Authors:** Aledie Navas Nazario, Felicia I Cooper, Fabiola Weber-Guzman, Richard S Finkel

**Affiliations:** 1 Pulmonology, Nemours Children's Health System, Orlando, USA; 2 Graduate Medical Education, Nemours Children's Health System, Orlando, USA; 3 Interventional Radiology, Nemours Children's Health System, Orlando, USA; 4 Neurology, Center for Experimental Neurotherapeutics - St Jude, Memphis, USA

**Keywords:** ullrich muscular dystrophy, respiratory failure, pneumothorax, non-invasive, blood patch

## Abstract

We present the case of a 19-year-old female with Ullrich congenital muscular dystrophy (UCMD1, a collagen VI defect) who developed a right-sided pneumothorax after choking on a piece of meat. She received two chest tubes (pigtails) that resolved the pneumothorax. She was discharged in stable condition, and a chest radiograph two weeks later showed total resolution of the pneumothorax. Two months after this episode, the patient presented with another small, right-sided pneumothorax that shortly progressed to extension throughout the right side of the chest. These pneumothoraces were treated with three different pigtails, but this intervention was ineffective. Providers chose to utilize an autologous blood patch, which is an injection of the patient’s own blood instilled in the pleural cavity through a chest drain. The blood forms a clot and subsequently seals the lung tissues through inflammation. This technique was chosen because the patient had advanced neuromuscular weakness with chronic respiratory failure. Also, our patient was not a candidate for chemical or surgical pleurodesis due to the nature of the persistent pneumothorax and the underlying lung fibrosis and collagen defect. Subsequent reaccumulation of the pneumothorax led to a second blood patch procedure, which proved effective. The patient recovered and was discharged in stable condition with no further episodes of pneumothorax over the subsequent 14 months from the initial episode.

## Introduction

There is a paucity of research regarding the development and treatment of pneumothorax in patients with Ullrich muscular dystrophy (UMD); most studies discuss conservative, chemical, or surgical pleurodesis to cure persistent pneumothorax. This is a challenging decision in the settings of severe restrictive lung disease and chronic respiratory failure, since these procedures require intubation and anesthesia (and thus place the patient at risk of ventilation dependence or death). Our case study of a 19-year-old with Ullrich congenital muscular dystrophy (UCMD) and persistent right-sided pneumothoraces highlights the option of a rarely utilized but effective treatment - an autologous blood patch.

## Case presentation

This patient was a 19-year-old non-ambulatory female with a history of UCMD who was diagnosed clinically at age 10 with genetic confirmation of pathogenic variants in the collagen VI A3 gene (COL6A3). Surgical correction of neuromuscular scoliosis was performed at age 11 years. Her pulmonary function testing prior to the acute presentation included forced vital capacity of 0.42 L, forced expiratory volume of 0.41 L/second, and peak cough flow of 2.22 L/sec. Routine care included nocturnal noninvasive positive pressure ventilation (NIPPV) with pressures of average volume-assured pressure support (AVAPS) tidal volume = 220, inspiratory positive airway pressure (IPAP) max = 22, IPAP min = 18, expiratory positive airway pressure = 5, rate = 20 breaths per minute, AVAPS rate = 5, inspiratory time = 1.0, and use of in/exsufflation ("cough assist") (Table 1).

**Table 1:** Pulmonary function testing chart with normal values.

	Patient’s value	Reference value	% Reference
Forced vital capacity	0.42 L	2.58 L (range: 2.1-3.1 L)	16
Forced expiratory volume in one second	0.41 L	2.23 L (range: 1.8-2.6 L)	18
Peak cough flow	2.22 L/sec	350 L/sec	0

A baseline chest radiograph demonstrated an asymmetric thorax with the right hemithorax having a parasol deformity and an elevated diaphragm on inspiration. The patient presented to the emergency department with unresolved chest pain and shortness of breath after choking on a piece of meat.

**Figure 1:**
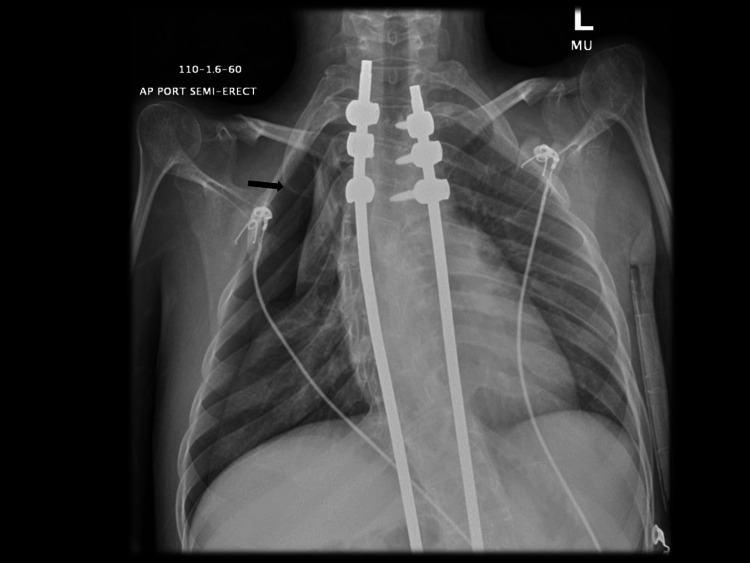
Chest radiograph of first pneumothorax located in the right upper lobe (black arrow).

A chest radiograph showed a right-sided pneumothorax (Figure 1).

**Figure 2:**
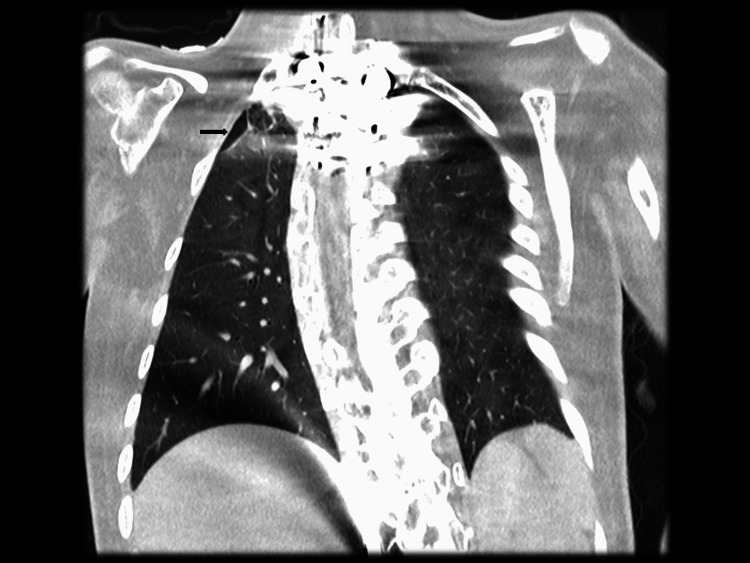
Coronal image of computed tomography scan showing partial resolution of right-side pneumothorax with residual pneumothorax on the right upper lobe (black arrow).

A pigtail chest tube was placed, and she was transferred to Nemours Children’s Health in Florida for admission. Upon admission, she developed respiratory distress, and a radiograph showed an extension of the pneumothorax requiring a second chest tube. She was continued on noninvasive positive pressure ventilation at her prior settings since she had severe restrictive lung disease and nocturnal respiratory insufficiency. Coronal computed tomography (CT) image of the chest showed partial resolution of the right-sided pneumothorax (Figure 2).

During this admission, several options were discussed, including chemical pleurodesis and a blood patch. The patient and her parents chose a conservative approach to treatment. The pneumothorax improved, and the patient was discharged home. A follow-up chest radiograph two weeks later showed total resolution of the pneumothorax.

**Figure 3:**
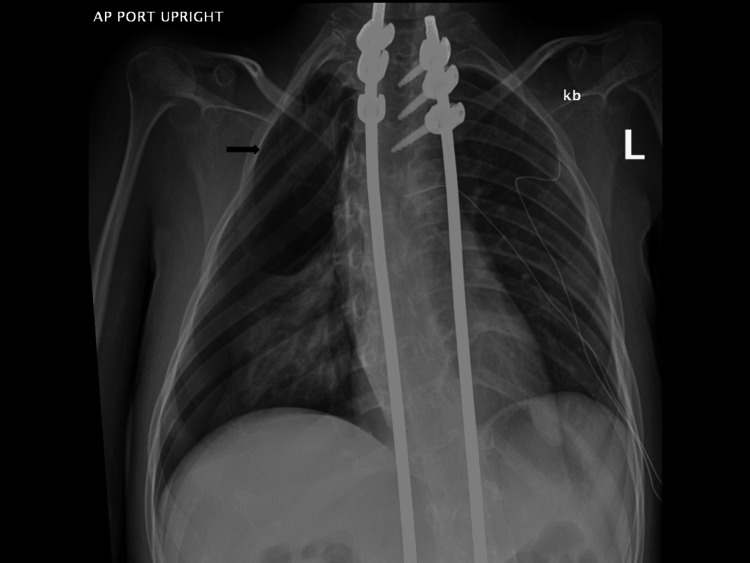
Chest radiograph of the second right-sided pneumothorax more extensive involving the right upper lobe (black arrow).

Two months after this first episode, the patient presented to a local emergency room with similar symptoms of shortness of breath and chest pain as before. A spontaneous right-sided pneumothorax was diagnosed, and a chest tube was placed (Figure 3).

**Figure 4:**
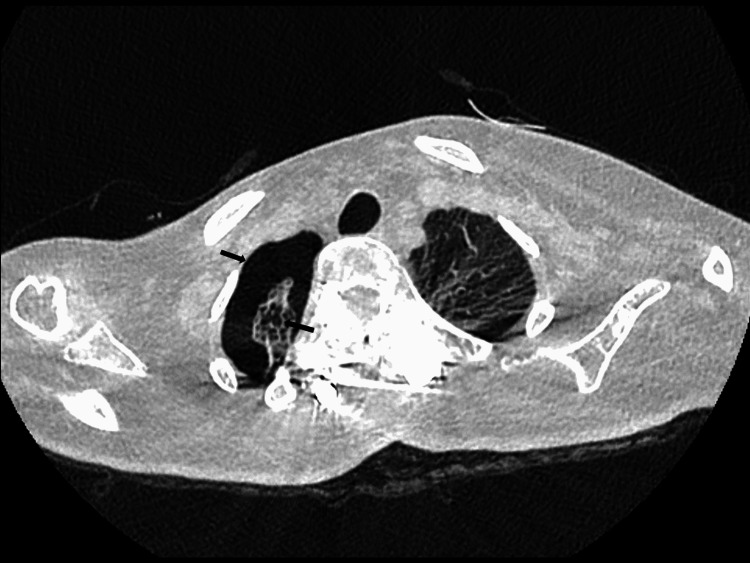
Axial computed tomography image shows multiple sub-centimeter cysts vs blebs in the right upper lobe parenchyma with pneumothorax (black arrows).

She was transferred to the intensive care unit at Nemours Children’s Health in Florida. Upon arrival, another chest radiograph confirmed the expansion of the pneumothorax. A second pigtail was placed with clinical improvement. During this admission, the patient remained on her baseline nocturnal noninvasive positive pressure ventilation as she had during the first admission. The patient subsequently required the third pigtail due to accumulation in the right basal area. This episode was not self-resolving as in the prior admission, and another CT scan was obtained, which showed multiple sub-centimeter cysts versus blebs in the right upper parenchyma with a pneumothorax (Figure 4).

The patient had several episodes of reaccumulation requiring repositioning of tubes and aspiration of air. Once the tubes were placed on a water seal, providers proceeded with a less-invasive technique: a blood patch. Since the pneumothorax was persistent and there was a risk of tension pneumothorax, the chest tube was not clamped. Instead, the chest tube was kept on a water seal and at a position above chest level so the backflow of blood into the drain was prevented. The patient was brought to the Trendelenburg position with the intention of targeting the right upper lobe. At the bedside in the intensive care unit, the venous blood of the patient was taken at a volume of 1 mL/kg (50 mL), and the collected blood was administered to the patient via the chest tube for over 30 minutes. A chest radiograph was obtained after the procedure, showing the blood patch in the right lung apex. 

**Figure 5:**
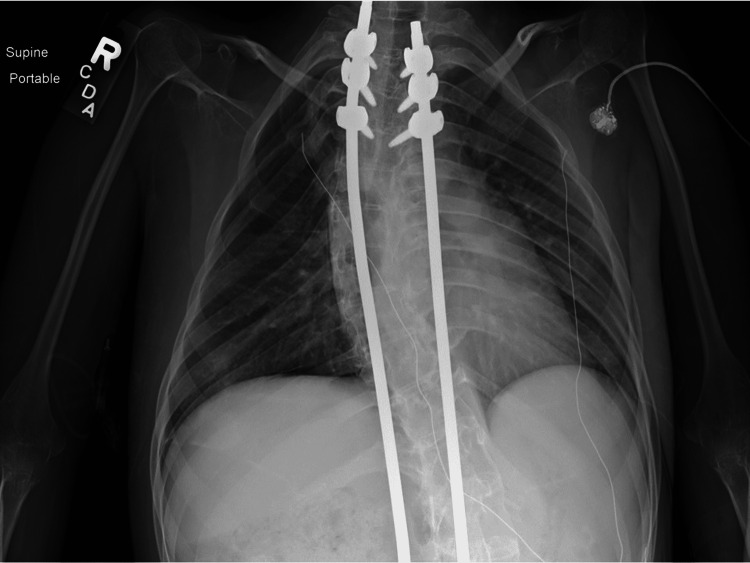
Last chest radiograph performed, showing almost complete resolution of the second pneumothorax after second blood patch.

Twenty-four hours after this procedure, the patient had clinical decompensation and developed a sensation of chest pressure. The chest radiograph showed reaccumulation. After the air was evacuated by repositioning of the posterior chest tube and aspiration of 200 ml of air, providers considered a second blood patch or surgical intervention but ultimately decided to administer another blood patch. The providers used the same protocol as for the first blood patch, and the patient tolerated the procedure well. She improved, and the chest tubes were clamped and subsequently removed one at a time. The anterior chest tube was removed 48 hours after the blood patch, and the posterior chest tube was removed four days after the procedure. She was discharged home on her baseline NIPPV settings, and her follow-up chest radiograph showed minimal pneumothorax at the apex that eventually resolved completely (Figure 5).

## Discussion

Ullrich congenital muscular dystrophy (OMIM 254090) is a collagen VI-related dystrophy in which there is mutation(s) in the COL6A1, COL6A2, or COL6A3 genes [1]. Collagen VI is widely distributed throughout extracellular matrices in various tissues and in the basement membranes of myofibers [2]. The condition usually has a dominant genetic mechanism. The clinical presentation includes distal hyperlaxity, proximal joint contractures, protruding calcanei, scoliosis, and respiratory insufficiency [2]. Consensus-based care recommendations have been published to guide clinicians [3].

In addition to composing skeletal muscle, collagen VI makes up the bronchial tree and alveoli and forms a connection between the lung extracellular matrix and basement membrane. The pathophysiology of how mutant collagen VI protein contributes to lung dysfunction is not completely understood [1]. The main causes of respiratory insufficiency in this patient were related to the development of scoliosis and respiratory muscle weakness and insufficiency (rather than a primary parenchymal lung disorder) [1,2]. Several cases of pneumothoraces in patients with UMD have been reported, and the mechanism is thought to be associated with increased predisposition to pleural blebs through loss of the structural integrity of the lung tissue [4].

To the best of our knowledge, no lung pathology in these collagen-VI-related disorders has been published. Patients with neuromuscular dystrophy and respiratory failure are often treated with non-invasive ventilation in addition to adjunctive therapies like lung volume recruitment and mechanical insufflation-exsufflation [5]. Some studies have found that these therapies "increase inspiratory capacity above maximal spontaneous inspiration" and, thus, increase the risk of pneumothoraces [6]. Since patients with UMD already have a predisposition to pneumothoraces because of the collagen defect, the addition of these pulmonary adjunctive therapies and positive pressure ventilation further increases the risk of pneumothorax. In their case report, Vianello et al. present two patients with Duchenne muscular dystrophy and pneumothorax after they received volume-targeted non-invasive positive pressure ventilation through nasal masks [7]. Choo-Kang et al. also describe a patient with an unidentified myopathy who developed "four distinct episodes of left-sided pneumothorax over a 12-month period; two of which occurred while he was using NIPPV support" [8]. Interestingly, the authors of the aforementioned article propose that their 26-year-old patient may have had Bethlem myopathy, an allelic collagen-type-VI disease, given his medical history and the appearance of numerous pleural blebs on lung imaging [8].

Fraser et al. presented two adults and three children with type-VI collagen defects who developed pneumothoraces [1]. However, these patients were treated with oxygen only (a method of conservative management, like our patient received during her first hospitalization), a thoracostomy tube, chemical pleurodesis, or open pleurodesis. Desikan et al. also discussed a 21-year-old patient with UCMD and two pneumothoraces (eight months apart) treated with intercostal drain insertion and chemical pleurodesis [9]. In our case, it was difficult to manage a pneumothorax according to the standard of care when the patient had severe restrictive lung disease and chronic respiratory failure (requiring ongoing positive-pressure non-invasive ventilation at night) in addition to the collagen defect.

Surgical treatment and pleurodesis are commonly performed to overcome a pneumothorax [1,10]. In chemical pleurodesis, agents such as talc powder, tetracycline derivatives, and bleomycin are used to eliminate the space between the parietal and visceral pleura and, thus, seal the air leak [10]. To achieve success with chemical pleurodesis, the lung must have begun to expand, and the pleural layers should be spaced close enough to allow adhesion [11]. In our patient’s case, the second pneumothorax was persistent, and the lung was non-expandable (i.e., she was not a candidate for chemical pleurodesis). 

The autologous blood patch procedure was first used in 1987 to repair persistent pneumothorax [12]. In this form of pleurodesis, blood is inserted through a patient’s chest tube and a clot begins to form [10]. Then, the fibrogenic activity of the blood generates pleurodesis by inducing pleural irritation and inflammation [13]. The blood clot obstructs the spaces in the lung parenchyma where the air leak occurs, stopping the air leak although the lung is non-expandable. Over time, this method prevents further episodes and improves lung expansion. In the literature, side effects and complications of the autologous blood patch procedure are significantly less when compared with chemical pleurodesis [10]. The main side effects reported are fever and empyema. Of note, the procedure is usually painless, and no sedatives or analgesics are needed [10]. In their research, Oliveira et al. found other studies with an 84-100% success rate for autologous blood patches for persistent air leaks, including their own success rate of 85% [4].

## Conclusions

In this patient with UCMD neuromuscular disease (due to a defect in collagen type VI) who presented with recurrent pneumothoraces that failed to respond to conservative first-line therapy, an autologous blood patch was proved effective. This procedure has a high efficacy and safety record and is generally well-tolerated. This article presents the successful outcome of a single patient; it can also represent a new treatment modality in patients with similar situations. This therapeutic option can be considered as a first-line treatment in patients with persisting pneumothorax and underlying collagen disorders (leading to chronic respiratory failure), especially when cystic lung lesions are present and when chemical pleurodesis and surgery are not suitable.
